# Home-School Cultural Value Mismatch: Antecedents and Consequences in a Multi-Ethnic Sample Transitioning to College

**DOI:** 10.3389/fpsyg.2021.618479

**Published:** 2021-09-06

**Authors:** Yolanda Vasquez-Salgado, Patricia M. Greenfield, Shu-Sha Angie Guan

**Affiliations:** ^1^Department of Psychology, California State University, Northridge, Northridge, CA, United States; ^2^Department of Psychology, University of California, Los Angeles, Los Angeles, CA, United States; ^3^Department of Child and Adolescent Development, California State University, Northridge, Northridge, CA, United States

**Keywords:** Latinx, first-generation college students, income, family obligation, cultural mismatch, cultural value mismatch, health, academic achievement

## Abstract

Qualitative work has documented *home-school cultural value mismatch*—a mismatch between collectivistic family obligations and individualistic academic obligations—experienced by Latinx first-generation college students during their first year of study at a 4-year university. This study extends prior research by examining home-school cultural value mismatch among a larger, multi-ethnic sample from Latinx, Asian and European American backgrounds (*N* = 155) in order to quantify the phenomenon and generalize it across multiple ethnic groups. Antecedents and consequences of different forms of mismatch were assessed in separate models. In modeling antecedents, we found that Latinx background, first-generation college status and low parental income were interconnected. However, among these three variables, it was first-generation college status that was the sole predictor of strong collectivistic motives for attending college; these motives were, in turn, associated with more frequent mismatch between family obligation and academic obligation. In addition, being female directly related to mismatch prevalence, as did living close to home. In modeling consequences of cultural value mismatch, frequent home-school cultural value mismatch predicted mental and physical health distress, which predicted academic problems; such problems were, in turn, related to lower grades. Our findings document the generalizability of this experience for first-generation college students from all ethnic backgrounds, as well as the unique experiences of students who identify as female or live close to home. Our findings also reveal the health and academic costs associated with this mismatch. Implications for research, intervention, and institutional change are discussed and have become increasingly important, given recent societal events that require most students to remain closer to home during distance learning.

## Introduction

Although enrollment of Latinx students in post-secondary institutions has increased, they continue to lag behind their Asian and European American peers in earning degrees (Solórzano et al., 2005; Pew Research Center, 2016). This pattern suggests that, while Latinx youth want to attend college, there are factors beyond college entrance that are deterring their academic success. Qualitative research suggests that Latinx first-generation college students (students whose parents did not earn a college degree) experience home-school cultural value mismatch (previously introduced as *home-school cultural value conflict*)— a mismatch between collectivistic family obligations and individualistic academic obligations (e.g., having to decide between attending a family event vs. studying)—during their transition to 4-year universities; the stress from this mismatch increases Latinx first-generation college students' vulnerability to mental, physical and academic distress (Vasquez-Salgado et al., 2015), all measures of overall college adjustment (Smedley et al., 1993). The purpose of the current study is to extend this body of work by surveying a larger, multi-ethnic sample, in order to quantitatively examine the antecedents and consequences of home-school cultural value mismatch and broaden generalizability to other groups.

### Theoretical Framework: Conceptualizing Sociodemographics and Cultural Values as Predictors of Home-School Cultural Value Mismatch

#### Cultural Mismatch Theory

Cultural Mismatch Theory and empirical research provide a foundation for the current research (Stephens et al., 2012a). Cultural Mismatch Theory was the first to document that health and educational disparities at the college level can be explained by differences in cultural dispositions (Stephens et al., 2012b). In the theory, first-generation college students (students whose parents did not receive a college degree) are more likely to experience *cultural mismatch*—mismatch between their collectivistic values (where priority is given to group goals and community cohesion) learned at home and the independent or individualistic values of 4-year institutions (where priority is given to personal needs and goals). This general sense of mismatch has been rigorously examined and documented as causing a disruption in academic performance for first-generation college students during the first-year of college. It also can disrupt health via an increase in the stress hormone, cortisol, when placed in an experimentally induced cultural mismatch situation (Stephens et al., 2012c).

Continuing-generation college students (students whose parents received a college degree) do not experience cultural mismatch because their individualistic values align with the values of the university culture. Though the theory is fruitful, more work is needed to pin-point the mechanisms involved in the antecedents and consequences of cultural mismatch at the college level. Our research addresses this issue by testing the mechanisms of one particular type of mismatch: home-school cultural value mismatch.

#### Theory of Social Change, Culture and Human Development: The Role of Education and Income

In a broader, complementary view, the Theory of Social Change, Culture and Human Development posits that sociodemographic factors influence cultural values, which are adaption to a particular social ecology (Greenfield, 2009). These sociodemographics and values then influence behavior. Hence, the theory posits multiple directional paths, from macro to micro, with a dominant direction of causality from sociodemographics to cultural values and, in turn, from cultural values to behavioral development.

In this framework, *collectivistic values* are an adaptation to ecologies in which formal education is limited and material resources are low. Empirical research confirms that low formal education and limited material resources are strongly linked to the development of collectivistic values (Kagitçibaşi, 2007; Park et al., 2014; Bianchi, 2016). In contrast, *individualistic values* are an adaptation to ecologies in which formal educational opportunity is great and material resources are more abundant. The theory proposes and empirical research confirms that a higher level of formal education and greater material resources are strongly linked to the development of more individualistic values (Kagitçibaşi, 2007; Park et al., 2014; Bianchi, 2016).

Similar to Cultural Mismatch Theory, the theory posits that movement from one social ecology (e.g., home) to another (e.g., university) can result in cultural mismatch (Greenfield, 2009). This mismatch can, in turn, influence development. Thus, we expect first-generation college status and low parent income to be correlated and serve as indicators that contribute to collectivism. However, we expect first-generation college status to be the stronger or even sole indicator of collectivistic motives for attending college, which was the aspect of collectivism that we assessed in the present research. We predict that higher levels of collectivism will result in more experiences with home-school cultural value mismatch during the transition to college. In this study, collectivism is defined as collectivistic motives for attending college because such motives have been previously utilized as markers of a general cultural mismatch (Stephens et al., 2012a).

#### Home-School Cultural Value Mismatch Among Latinx Youth Across Development

Research conducted with Latinx families from socioeconomically disadvantaged homes has found evidence for home-school cultural value mismatch at different developmental stages. Early in development, the mismatch manifests as a discrepancy between parent and teacher values (Trumbull et al., 2001; Greenfield and Quiroz, 2013). This mismatch becomes internalized in high school where Latinx youth begin to feel torn between choices of working to help their families or going to school (Suárez-Orozco and Suárez-Orozco, 1995). However, the existence of such mismatch, once students have made a commitment to higher education, has only been recently examined (Vasquez-Salgado et al., 2015, 2018). Those studies point to negative consequences of home-school cultural value mismatch for health and academic development of Latinx first-generation college students during the transition to a 4-year university (*See Consequences of Home-School Cultural Value Mismatch* for further details).

Continued examination of this form of mismatch during the transition to university is crucial because longitudinal research suggests that family obligation (e.g., attending family events, assisting family), a collectivistic value, increases during emerging adulthood (Fuligni et al., 1999; Fuligni and Pedersen, 2002), a period in development that typically overlaps with entry to university. The present research extends prior work (Greenfield, 2009; Stephens et al., 2012a) by incorporating Latinx ethnicity, first-generation college status and income into one model that tests for the antecedents of cultural mismatch.

We expect these sociodemographic variables (Latinx ethnic background, first-generation college status, income) to correlate with one another because research conducted at various 4-year universities across the nation suggest that Latinx youth are more likely to be first-generation college students (Saenz et al., 2007) and come from households with limited financial resources (Guan et al., 2014). Research also suggests that first-generation college students typically come from low-income households (Terenzini et al., 1996; Horn and Nunez, 2000).

Moreover, given prior research and theory, we expect that in the context of all sociodemographic variables, first-generation college status will be the key variable linked to collectivism, and higher levels of collectivism will predict more experiences with mismatch. If results are as expected, the scientific community will be able to generalize the experience of home-school cultural value mismatch to first-generation college students from other ethnic backgrounds, as the key mechanism is expected to be first-generation college status and cultural values rather than ethnicity.

### Additional Sociodemographic Predictors of Home-School Cultural Value Mismatch

#### Geographical Distance From One's Family Home

Available studies suggest that not all Latinx first-generation students experience home-school value mismatch in a similar way. Qualitative research indicated that living closer to one's family yields more home-school cultural value mismatch experiences (e.g., having to decide between visiting family and studying; Vasquez-Salgado et al., 2015). Experimental research showed that such mismatch disrupts the attentional focus of Latinx first-generation college students—but does so only for students living close to home (Vasquez-Salgado et al., 2018). These findings align with findings that students living in closer proximity to their parents' homes not only have stronger family obligation values (Fuligni and Pedersen, 2002), but also enact such obligations behaviorally to a greater extent (Tseng, 2004). We extend prior research by testing a direct quantitative relation between geographical distance from home and home-school cultural value mismatch in a multi-ethnic sample.

#### Gender

An experiment demonstrated that females (particularly those living close to home) respond to a home-school cultural value mismatch condition with greater attentional disruption than do males (Vasquez-Salgado et al., 2018). This finding aligns with prior findings that females are more heavily burdened by family obligations than are males (Stein et al., 2014). The finding is also in accord with the idea that traditionally ascribed gender roles require females to fulfill productive tasks at home and males to fulfill such tasks further from home (Manago et al., 2014); the family obligations that are central to home-school cultural value mismatch involve family obligations that typically occur at home (i.e., spending time with family, attending family events, assisting family). The current research adds to the existing knowledge base by testing a direct quantitative relation between gender and experiences with home-school cultural value mismatch in a multi-ethnic gender-balanced sample.

### Hypotheses About Antecedents of Home-School Cultural Value Mismatch

**Figure 1:**
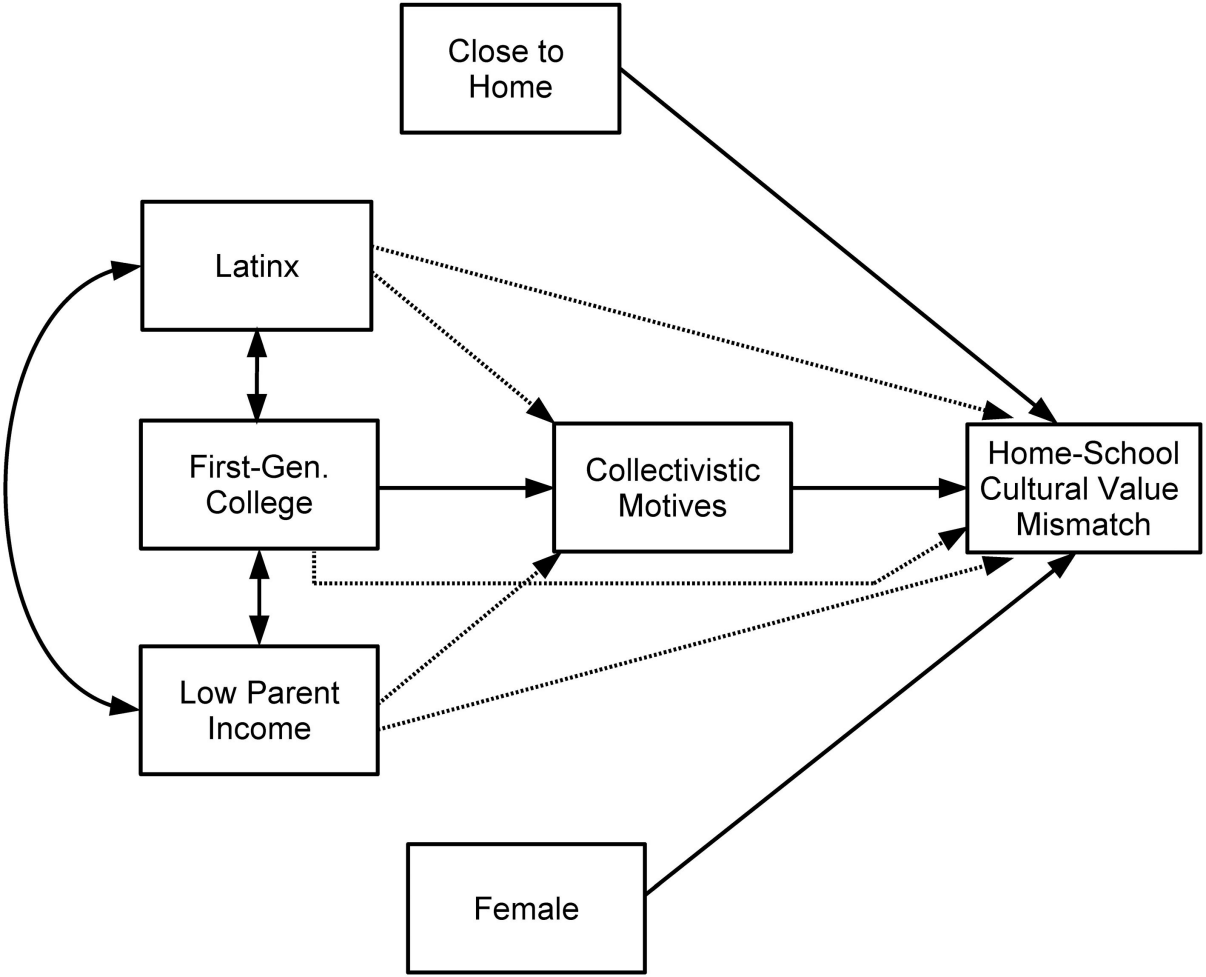
Hypothesized model for antecedents of home-school cultural value mismatch. A dashed line indicates a non-significant relation was expected within the model. Latinx = Latinx ethnic background (coded as 1) vs. not (coded as 0); First-Gen. College = First-generation college student (students' parents did not receive a college degree; coded as 1) vs. continuing-generation college student (coded as 0); Female = Female (coded as 1) vs. male (coded as 0).

Based on the aforementioned theories and empirical work, we propose the following hypotheses (Figure 1) regarding the antecedents of home-school cultural value mismatch. If confirmed, this will yield greater understanding of the role that sociodemographics and cultural values play in students' experiences with this form of mismatch during the transition to college.

**Hypothesis 1:** There will be significant intercorrelations between Latinx ethnic background, first-generation college status and low parent income (Figure 1).
**Hypothesis 2:** However, among these three sociodemographic variables, first-generation college status will be the only variable that predicts strong collectivistic motives for attending college (Figure 1).
**Hypothesis 3:** Strong collectivistic motives will then predict more experiences with home-school cultural value mismatch (Figure 1).
**Hypothesis 4:** Moreover, because we expect cultural values to serve as the main mediator linking first-generation college status and frequency of home-school cultural value mismatch, a direct relation between these two variables was not expected to be present within the larger model (Figure 1). Instead, the hypothesis is that first-generation college status will have a significant indirect effect on frequency of home-school cultural value mismatch through collectivistic motives for attending college (Figure 1).
**Hypothesis 5:** Lastly, identifying as female and living closer to home will also directly predict more experiences with home-school cultural value mismatch (Figure 1).

It is important to note that by including geographical distance from home and gender in our model, we tested for direct relations of these variables with home-school cultural value mismatch; at the same time, their inclusion enabled us to control for these variables.

### Consequences of Home-School Cultural Value Mismatch

Our basic theorem is that home-school cultural value mismatch, that is, mismatch between collectivistic family obligations and individualistic academic obligations, produces inner turmoil and negative overall adjustment (Greenfield, 2009; Stephens et al., 2012a,c; Vasquez-Salgado et al., 2015, 2018). We relied on prior research, especially students' subjective experiences with this mismatch, to form the basis of our expectations regarding the particular pathway by which home-school cultural value mismatch relates to overall adjustment.

#### Home-School Cultural Value Mismatch as a Predictor of Mental and Physical Health Distress

Individuals with a collectivistic orientation experience psychological distress when in an individualistic setting (Caldwell-Harris and Ayçiçegi, 2006; Stephens et al., 2012c). Most pertinent to our study, this pattern has been observed at the college level via physiological measures (i.e., increases in cortisol; Stephens et al., 2012c) and subjective reports of distress among first-generation college students (Vasquez-Salgado et al., 2015). Several Latinx first-generation college students in Vasquez-Salgado et al.'s (2015) sample reported distress when confronting home-school mismatch during their first year in college (i.e., stress, guilt, emotionality; perceived negative impact on physical health). We therefore predict that a higher frequency of home-school cultural value mismatch will relate to reports of more mental and physical health distress.

#### Mental and Physical Health Distress as a Predictor of Academic Problems

Correlational and experimental studies suggest that individuals who experience psychological distress are also prone to experiencing problems with regulating their attention (Mathews and MacLeod, 1985; Liston et al., 2008; Yiend, 2010; Meyers et al., 2014) and engaging in learning activities (Ramirez and Beilock, 2011; Rozek et al., 2019). Central to the current study, first-generation college students expressed in focus-group conversations that they were unable to concentrate or study because of the distress they experienced from home-school cultural value mismatch (Vasquez-Salgado et al., 2015). This lack of concentration was often coupled with other school problems, such as not understanding material and not completing assignments. There were also indications that these academic problems lowered grade-point average. Therefore, we expect that distress from home-school cultural value mismatch will relate to academic problems surrounding attention and learning; these problems will be associated with low grade-point average.

#### Hypotheses About Consequences of Home-School Cultural Value Mismatch

**Figure 2:**
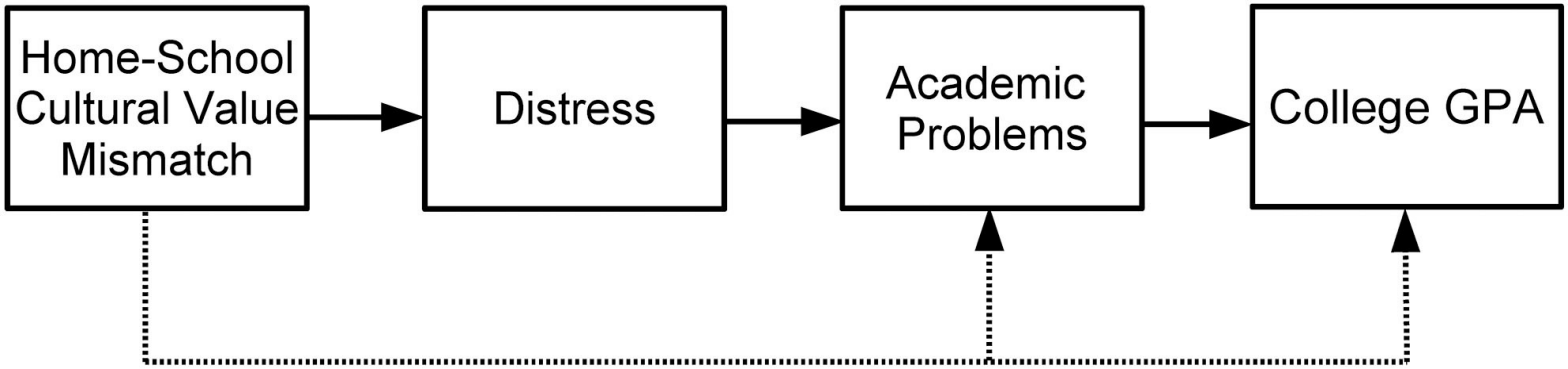
Hypothesized model for consequences of home-school cultural value mismatch. A dashed line indicates a non-significant relation was expected.

Based on the aforementioned theoretical perspectives and empirical work, we propose the following path model (Figure 2) about consequences of home-school cultural value mismatch. If confirmed, this model will provide a quantitative step toward understanding the intrapersonal mechanisms that unfold as a consequence of experiencing cultural value mismatch in the transition to college.

**Hypothesis 6a:** Integrated into one large model (Figure 2), home-school cultural value mismatch will relate to mental and physical health distress.
**Hypothesis 6b:** Distress will, in turn, be associated with academic problems surrounding attention and learning (Figure 2).
**Hypothesis 6c:** Academic problems will then be linked to low college GPA (Figure 2).
**Hypothesis 6d:** In addition, because we expect distress to serve as the main mediator that links home-school cultural value mismatch to academic problems, a direct link from mismatch to academic problems was not expected to be present within the model once distress was accounted for (Figure 2). Instead, the hypothesis is that home-school cultural value mismatch will have a significant indirect effect on academic problems through distress (Figure 2).
**Hypothesis 6e:** Lastly, because we expect distress and academic problems to serve as the main mediators that link home-school cultural value mismatch to GPA, a direct link from mismatch to GPA was not expected to be present within the model after accounting for distress and academic problems (Figure 2). Instead, the hypothesis is that home-school cultural value mismatch will have a significant indirect effect on GPA through the paths of the intervening variables (Figure 2).

## Method

### Participants

Toward the end of their first year of college at the University of California, Los Angeles (UCLA), a large, public, research university in Southern California, students were recruited via the psychology subject pool, flyers posted throughout campus, and direct emails. The only criterion to join the study was that participants be in their first year of study at the institution and age 18 or older. There were 10 transfer students in the final sample. The average age of our final sample was 18.91 (*SD* = 1.40). The average age of our final sample was 18.91 (*SD* = 1.40) and 22.5% were psychology or psychobiology majors.

Across 2-years, a total of 186 students completed the survey. Previous studies focusing on the topic of family obligation have largely centered on Latinx, Asian American, and European American groups (e.g., Fuligni et al., 1999; Fuligni and Pedersen, 2002; Telzer and Fuligni, 2009). These were also the three main groups present at the research site. Thus, we limited our sample to these groups. This decision decreased our sample size to 155. It is important to mention that no previous quantitative study exists to help determine the number of participants necessary as the concepts we are investigating are novel and have only been explored qualitatively. Therefore, no power analysis was conducted. Our team collected as much data as possible across 2-years.

Sample size was fairly equal across the three ethnic groups (Asian = 46; European American = 49; Latinx = 60). Note that, although 45.2% of our sample were first-generation college students, most were from Latinx and Asian backgrounds; there were very few European American first-generation college students. In fact, a chi-square test revealed that Latinx students (*n* = 52) were first-generation college students significantly more often than their Asian (*n* = 14), χ^2^(1, *N* = 106) = 35.04, *p* < 0.001, and European American peers (*n* = 4), χ^2^(1, *N* = 109) = 66.54, *p* < 0.001. In addition, Asian students were first-generation college students significantly more often than European American students, χ^2^(1, *N* = 95) = 7.66, *p* = 0.006.

There was also a significant difference among the various ethnic groups in reported parental income, *F*_(2,152)_ = 32.45, *p* < 0.001. Latinx students (*M* = 4.87, *SD* = 2.88) reported significantly lower parent income than did those from Asian (*M* = 7.60, *SD* = 3.78) and European American (*M* = 9.87, *SD* = 3.11) backgrounds, *t*_(81.74)_ = 4.30, *p* < 0.001, and *t*_(99.22)_ = 8.63, *p* < 0.001, respectively. The income reported by students from Asian backgrounds was also significantly lower than that reported by European Americans, *t*_(87.36)_ = 3.18, *p* = 0.002. These socioeconomic differences in parental education and income are in line with the pattern of ethnic group differences across 4-year universities (Saenz et al., 2007) as well as at the site of the current research (Guan et al., 2014).

Lastly, our sample was equally distributed in gender (51% female) and the majority of students in our sample lived in campus dormitories (93.5%, *n* = 145). A few students reported that they lived with their parents (2.6%, *n* = 4), off-campus with friends (2.6%, *n* = 4) or other living situation (1.3%, *n* = 2). Those who reported an “other” living situation indicated that they lived “off-campus.”

### Procedure

Participants were told that a purpose of the study was to explore the nature and developmental consequences of home-school relations during the first year of college at the institution. After giving consent, participants completed the 25–30 min survey. Participants received research participation credits, a movie ticket, or $20 as compensation. All procedures were approved by the Institutional Review Board.

### Measures

#### First-Generation College Student Status

Students who reported that their parents' highest level of education was “some college or less” were considered “first-generation college students (coded as 1).” Those that reported that at least one of their parents had “a Vocational or A.A. degree” or higher were considered “continuing-generation college students (coded as 0).” In other words, participants were considered first-generation college students as long as their parents had not received any type of degree. It is noteworthy to mention that though there are variations in the definition of first-generation college status across the literature (Saenz et al., 2007; Stephens et al., 2012a; Toutkoushian et al., 2019), we decided on this particular operational definition as it is aligned with our most recent publication on home-school cultural value mismatch (Vasquez-Salgado et al., 2018).

#### Parent-Income

Students were asked, “How much is your parents combined yearly income?” Responses ranged from 1 = *Less than $10,000*, to 12 = *More than $150,000*.

#### Collectivistic Motives for Attending College

A 6-item measure assessing the extent to which students held collectivistic motives for attending college was utilized (Stephens et al., 2012a). Sample items included: “I decided to attend college so that I can…” “Help my family out after I'm done with college,” and “Bring honor to my family.” Participant responses ranged from 1 = *Strongly disagree* to 5 = *Strongly agree*. The items yielded a Cronbach's alpha of 0.87.

#### Home-School Cultural Value Mismatch

A 14-item measure assessing mismatch between the behavioral enactment of collectivistic family obligations and individualistic academic obligations was created. The measure was originally constructed to include five types of home-school cultural value mismatch. Three of these types were mismatch that came up during a focus group study of Latinx first-generation college students (Vasquez-Salgado et al., 2015): attending family events vs. doing academic work (3-items), spending time with family vs. doing academic work (3 items), and assisting family vs. doing academic work (4-items). Two additional types of mismatch were based on Fuligni et al.'s (1999) measure of family obligation: living further from home vs. remaining close to home (2-items), using money to help family vs. for college expenses (2-items). Sample items included: “Since you started UCLA, how often have you had to choose between doing your academic work and…” “attending family events,” “spending time at home with your family” and “helping take care of family members.” Responses ranged from 1 = *Never* to 4 = *Frequently*. The Cronbach's alpha for all items was 0.90.

An exploratory principal components analysis with promax rotation was used to identify factors that were assumed to be correlated in the novel 14-item scale. The factor analysis resulted in a Kaiser-Meyer-Olkin (KMO) coefficient of 0.846 and Bartlett's Test of Sphericity χ^2^ (91) = 1,400, *p* < 0.001, indicating significant sample adequacy. Three factors emerged from the scree plot with Eigenvalues >1.00. As shown in Appendix A, this three-factor solution included Factor 1: events and time with family vs. academics (with the exception of holidays; e.g., “choose between…academic work and…attending family events”); Factor 2: family assistance vs. academics, based on prior cultural mismatch work (e.g., “choose between…academic work and…doing tasks your family needs done”; Vasquez-Salgado et al., 2015); and Factor 3: long-term family obligations vs. academics, such as remaining close to home and using money to help family based on the broader family obligation literature (e.g., “choose between using your money for college expenses…and giving your family money for something they need”; Fuligni et al., 1999) explained 67.99% of the variance.

Based on the factor analysis, the hypothesized models were tested separately for each of the three home-school cultural value mismatch factors. It is important to clarify that the term, long-term family obligations vs. academics, was coined to describe factor 3 because mismatch situations that were included in that factor appeared more definitive in one's choice (between academics and family) and thus, likely to have long-term consequences, such as not having money for college expenses or seeing one's family not have resources they need. These types of situations may have the potential for devastating consequences, more so than typical forms of family assistance (e.g., completing one's academic work vs. helping one's brothers or sisters with their homework). In addition, we believe that decisions around choosing between engaging in academic work or spending holidays with family mapped onto factor 3 because it too has long-term consequences; not spending a holiday with family, more so than casual events or occasionally helping with light chores, may potentially leave a long-lasting imprint. This imprint is in hallmark memories created or lost or in photos from holidays shared among family members as described in Vasquez-Salgado et al. (2015). Finally, another mismatch situation that mapped onto this sub-scale was wanting to go further away from home but family members wanting students to continue living at home or attend a college closer to home. It is important to note that this form of mismatch could occur for a student that makes either decision (e.g., those who choose to go further away from home or continue to live at home). The idea is that their choice has long-term consequences as one cannot easily shift one's decision and thus, may continue to grapple with their choice in the long-term (e.g., frequent regret in not having moved away or guilt experienced in having moved away from home; as described in Vasquez-Salgado et al., 2015). Nonetheless, given our prior work, it is expected that all forms of home-school cultural value mismatch (Factors 1 through 3) would occur more frequently among students living geographically closer to home as family obligations are expected to be more salient and proximal, and thus, more likely to mismatch with academic obligations.

#### Mental and Physical Health Distress

A combination of 19-items was utilized to capture students' feelings of mental and physical health distress since they started at UCLA. Seven-items were taken from Huynh and Fuligni's (2010) adapted version of Lorr and McNair's (1971) Profile of Mood States. Students were asked to rate the extent (1 = *Not at all* to 5 = *Extremely*) to which they felt “on edge,” “nervous,” “uneasy,” “unable to concentrate,” “sad,” “hopeless” and “discouraged.” The remaining 12-items were taken from Huynh and Fuligni's measure of physical complaints that was created from existing measures. Using a 5-point scale (1= *Not at all*; 5 = *Almost every day*), participants rated the extent to which they experienced a set of physical complaints. Examples of complaints included, “headaches,” “very tired,” “dizziness,” “stomachaches” and “poor appetite.” Together, all items, previously utilized with diverse samples (Chung et al., 2009; Huynh and Fuligni, 2010; Huynh, 2012), yielded an alpha of 0.92.

The only change from the original items was to direct participants' attention to their time at the institution by prefacing the instrument with “Since you started UCLA.” In terms of calculation, in order to provide equal weight to mental and physical distress items, mental health (7-items) and physical health items (12-items) were first averaged separately, and, thereafter, both of these averages were combined to form one average.

#### Academic Problems

A 6-item measure of academic problems was utilized. Students were asked to rate (1 = *Never* to 5 = *Always*) how often they experienced certain situations since they started at the institution. These situations included attention (3-items; e.g., “had a difficult time focusing on studying”) and learning problems (3-items; e.g., “did not turn in homework that was due”; adapted from Telzer and Fuligni, 2009). All items reflected students' attention and learning difficulties that had come up in focus groups with first-generation college students from Latinx families (Vasquez-Salgado et al., 2015). Together, all items, previously utilized with a diverse sample (Telzer and Fuligni), yielded an alpha of 0.83. The only change from the original items was to direct participants' focus to their time at the institution by prefacing the instrument with “Since you started UCLA.”

#### College GPA

Students' self-reports of their grade-point average (GPA) earned in the Fall and Winter quarter of their first year at the institution were assessed using a scale ranging from 0.00 to 4.00. The GPAs of both quarters were combined to form one overall average.

#### Geographical Distance From Home

Geographical distance utilized an ordinal scale to capture the distance that students lived from their family's home. Students were asked, “If you decide to travel home, how long will it take you (e.g., 30 min, 1 h)?” Their response to this question was initially converted into minutes. Thereafter, in order to account for students who lived with family members and those who had parents living outside the country, new, ordinal codes were created. If a student indicated that they lived with their parents or other relatives, their distance was coded as “0.” If a student indicated that their parents lived outside the country, their distance was coded as “7.” Codes for the distances between these values were as follows: “1” (45 min or less), “2” (50 to 60 min), “3” (75 to 105 min), “4” (120 to 210 min), “5” (240 to 480 min; most of these participants had parents that lived in-state but far away), and “6” (519 to 2,400 min; mostly those with families living out of state).

#### Gender

Students' gender was assessed by their response to one question: “What is your gender?” Response were coded as 1 = female and 0 = male.

### Design and Analysis

Two separate processes were examined: antecedents and consequences of home-school cultural value mismatch. Path analysis, a structural equation modeling (SEM) technique, using Maximum likelihood (ML) estimation in EQS 6.1 for Windows, was utilized. Several studies have recommended 100 to 150 participants as the minimum sample size for structural equation models Ding et al. (1995) and this is consistent with our sample size (*N* = 155). Additionally, Bentler (2006) suggested that a saturated model with *p* variables has *p*(*p* + 1)/2 free parameters to be estimated. In the current study, there were *p* = 7 observed variables in the antecedents model, for example, resulting in 28 parameters to be estimated. Bentler and Chou (1987) suggested a sample size of 5–10 participants to number of free parameters and this is consistent with our sample size (28 ×5 = 140). In the consequences model, there were *p* = 4 observed variables resulting in 10 free parameters to be estimated (10 × 5 = 50). Because we did not have the sample size to combine all the variables into one model (55 parameters × 5 = 275), we examined the paths in two separate models. Thus, two models rather than an integrated one were created based on the number of estimated parameters recommended in a model as a function of sample size (Bentler and Chou, 1987; Bentler, 2006). The hypothesized antecedents (Figure 1) and consequences (Figure 2) models were based on theoretical considerations; separate models were tested for each of the three home-school cultural value mismatch factors that emerged from our data. Model fit was evaluated using chi-square (*x*^2^), comparative fit index (CFI), and root mean square error approximation (RMSEA). The model is a “good” fit if the *x*^2^ is not significant or near non-significance, the CFI is ≥0.95, and RMSEA is ≤0.05 (Byrne, 2006). The model is of “moderate” fit when at least two of these elements are met (Byrne, 2006; Vasquez-Salgado and Chavira, 2014). If the fit was appropriate, direct and indirect effects were examined. A direct effect is when one variable significantly predicts another, and an indirect or mediated effect is when one variable predicts another variable through one or more intervening variables (Kline, 2011). In order to confirm whether an indirect effect was the main source of influence, a direct path between those variables must not be statistically significant. If the direct path is significant, this implies that the indirect effect explains only part of the relation between one variable and another; non-significance indicates full mediation, and thus, that the indirect effect fully explains the relationship (Kline).

## Results

### Preliminary Analyses

Means, standard deviations, and zero-order correlations for all variables of interest are presented in Tables 1, 2. All expected relations between the variables were as predicted or in the expected direction. The data were also screened for normality. Examination of the skewness and kurtosis indices indicated that there were no values over 3.0 (Bentler, 2006).

**Table 1:** Zero-order Correlations for Variables of Interest in Models Assessing Antecedents of Home-School Cultural Value Mismatch.

	**1**.	**2**.	**3**.	**4**.	**5**.	**6**.	**7**.	**8**.	**9**.
1. Latinx	1								
2. First-Gen. College Status	0.66^***^	1							
3. Parent Income	−0.50^***^	−0.69^***^	1						
4. Collectivistic Motives	0.42^***^	0.47^***^	−0.43^***^	1					
5. Distance	−0.42^***^	−0.28^***^	0.16^+^	−21^**^	1				
6. Gender	0.01	0.14^+^	−0.11	0.05	−0.10	1			
7. Events and Time with Family vs. Academics	0.23^**^	0.23^**^	−0.15+	0.35^***^	−0.31^***^	0.25^***^	1		
8. Family Assistance vs. Academics	0.21^**^	0.23^**^	−0.19^*^	0.32^***^	−0.22^**^	0.27^***^	0.65^***^	1	
9. Long-term Family Obligations vs. Academics	0.41^***^	0.45^***^	−0.49^***^	0.47^***^	−0.18^*^	0.20^*^	0.38^***^	0.44^***^	1
Mean	0.39	0.45	7.26	3.84	3.51	0.51	2.58	2.16	2.18
Stand. Dev.	0.49	0.50	3.85	0.91	1.88	0.50	0.89	1.00	0.86

*Latinx, Latinx ethnic background (coded as 1) vs. not (coded as 0); First-Gen. College Status, First-generation college student (coded as 1) vs. continuing-generation college student (coded as 0); Gender, Female (coded as 1) vs. male (coded as 0);*

*** *p < 0.001*,

** *p < 0.01*,

* *p < 0.05*,

+ *p < 0.10*.

**Table 2:** Zero-order Correlations for Variables of Interest in Models Assessing Consequences of Home-School Cultural Value Mismatch.

	**1**.	**2**.	**3**.	**4**.	**5**.	**6**.
1. Events and Time with Family vs. Academics	1					
2. Family Assistance vs. Academics	0.65^***^	1				
3. Long-term Family Obligations vs. Academics	0.38^***^	0.44^***^	1			
4. Mental and Physical Health Distress	0.30^***^	0.26^***^	0.32^***^	1		
5. Academic Problems	0.24^**^	0.22^**^	0.25^**^	0.58^***^	1	
6. College GPA	−0.08	−0.13	−0.23^**^	−0.21^**^	−0.40^***^	1
Mean	2.58	2.16	2.18	2.45	2.73	3.24
Stand. Dev.	0.89	1.00	0.86	0.74	0.64	0.57

*** *p < 0.001*,

** *p < 0.01*,

* p < 0.05.

### Antecedents of Home-School Cultural Value Mismatch: Overall Models

**Figure 3:**
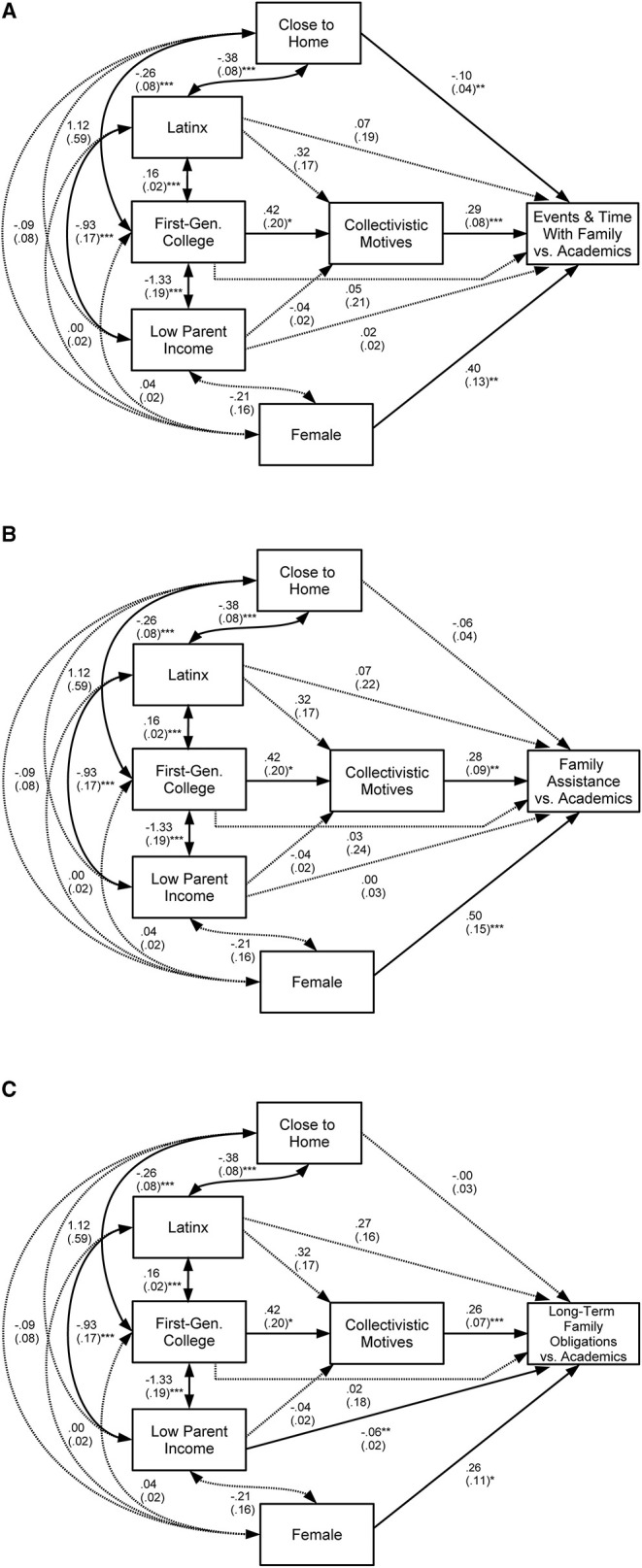
Antecedents of the three forms of home-school cultural value mismatch: **(A)** events and time with family, **(B)** family assistance, and **(C)** long-term family obligations vs. academics. Included are unstandardized estimates (with standard errors); a solid line indicates significance and a dashed line indicates non-significance; ***p < 0.001, **p < 0.01, *p < 0.05.

The models testing the antecedents of home-school cultural value mismatch, in terms of events and time with family [χ^2^(2, *N* = 155) = 0.64, *p* = 0.727, CFI = 1.00, RMSEA = 0.00; Figure 3A], family assistance [χ^2^(2, *N* = 155) = 0.64, *p* = 0.727, CFI = 1.00, RMSEA = 0.00; Figure 3B], and long-term family obligations [χ^2^(2, *N* = 155) = 0.64, *p* = 0.727, CFI = 1.00, RMSEA = 0.00; Figure 3B] during the first year of college fit the data well. Overall, 26% of the variance in collectivistic motives for attending college, and 19–36% of the variance in home-school cultural value mismatch was explained by the three models.

Table 1 shows that Latinx ethnicity, first-generation college status, and low parent income are all associated with each of the three types of cultural mismatch. However, we will show through our path analysis models that first generation college status is key in the mechanism underlying experiences with mismatch.

**Hypothesis 1**. There will be significant intercorrelations between Latinx ethnicity, first generation college status and low parent income (Figure 1). As Table 1 and Figures 3A–C show, this hypothesis was confirmed. The intercorrelations held for all three aspects of home-school cultural value mismatch.

**Hypothesis 2**: However, among these three sociodemographic variables, first-generation college status will be the only variable that predicts strong collectivistic motives for attending college (Figure 1). This direct path was confirmed for all three aspects of home-school cultural value mismatch (Figures 3A–C).

**Hypothesis 3:** Strong collectivistic motives will then predict more experiences with home-school cultural value mismatch (Figure 1). This direct effect was confirmed for all three aspects of home-school cultural value mismatch (Figures 3A–C).

**Hypothesis 4:** Moreover, because we expect cultural values to serve as the main mediator linking first-generation college status and frequency of home-school cultural value mismatch, a direct relation between these two variables was not expected to be present within the larger model (Figure 1). Instead, the hypothesis is that first-generation college status will have a significant indirect effect on frequency of home-school cultural value mismatch through collectivistic motives for attending college (Figure 1).

As expected, after accounting for Latinx background, parental income, and collectivistic motives, first-generation college status did not relate significantly to any of the three types of mismatch (Figures 3A–C). Instead, there was a marginally significant indirect effect linking first-generation college status background to the frequency of all components of home-school cultural value mismatch through the mediating variable of collectivistic motives (events and time with family: unstandardized indirect effect = 0.12; standardized indirect effect = 0.07; standard error = 0.07, *p* = 0.075; family assistance: unstandardized indirect effect = 0.12; standardized indirect effect = 0.06; standard error = 0.07, *p* = 0.087; long-term family obligations: unstandardized indirect effect = 0.11; standardized indirect effect = 0.06; standard error = 0.06, *p* = 0.073). Note also that the link between first-generation college status and collectivistic motives for attending college is statistically significant, as is the link between collectivistic motives for attending college and the three forms of home-school cultural value mismatch (Figures 3A–C).

Further demonstrating the central nature of first-generation college status and collectivism, Latinx ethnic background and parental income did not significantly predict home-school cultural value mismatch pertaining to events and time with family and family assistance (Figures 3A,B). However, the model pertaining to home-school cultural value mismatch related to long-term family obligations (Figure 3C) was slightly different. Specifically, though there were not any ethnic differences (i.e., Latinx background relative to other ethnic backgrounds), lower parental income directly related to frequency of this form of home-school cultural value mismatch (Figure 3C).

**Hypothesis 5:** Lastly, Identifying as female and living closer to home will directly predict more experiences with home-school cultural value mismatch. In terms of gender differences, this hypothesis was confirmed for all three aspects of home-school cultural value mismatch (Figures 3A–C).

Living close to one's family home predicted home-school cultural value mismatch in terms of grappling with decisions around attending events and spending time with family vs. focusing on one's academics (Figure 3A). Note also that geographical distance from home was significantly correlated with Latinx background and first-generation college status (Figures 3A–C); this relation implies that Latinx and first-generation college students generally tended to live closer to home.

### Consequences of Home-School Cultural Value Mismatch: Overall Models

**Figure 4:**
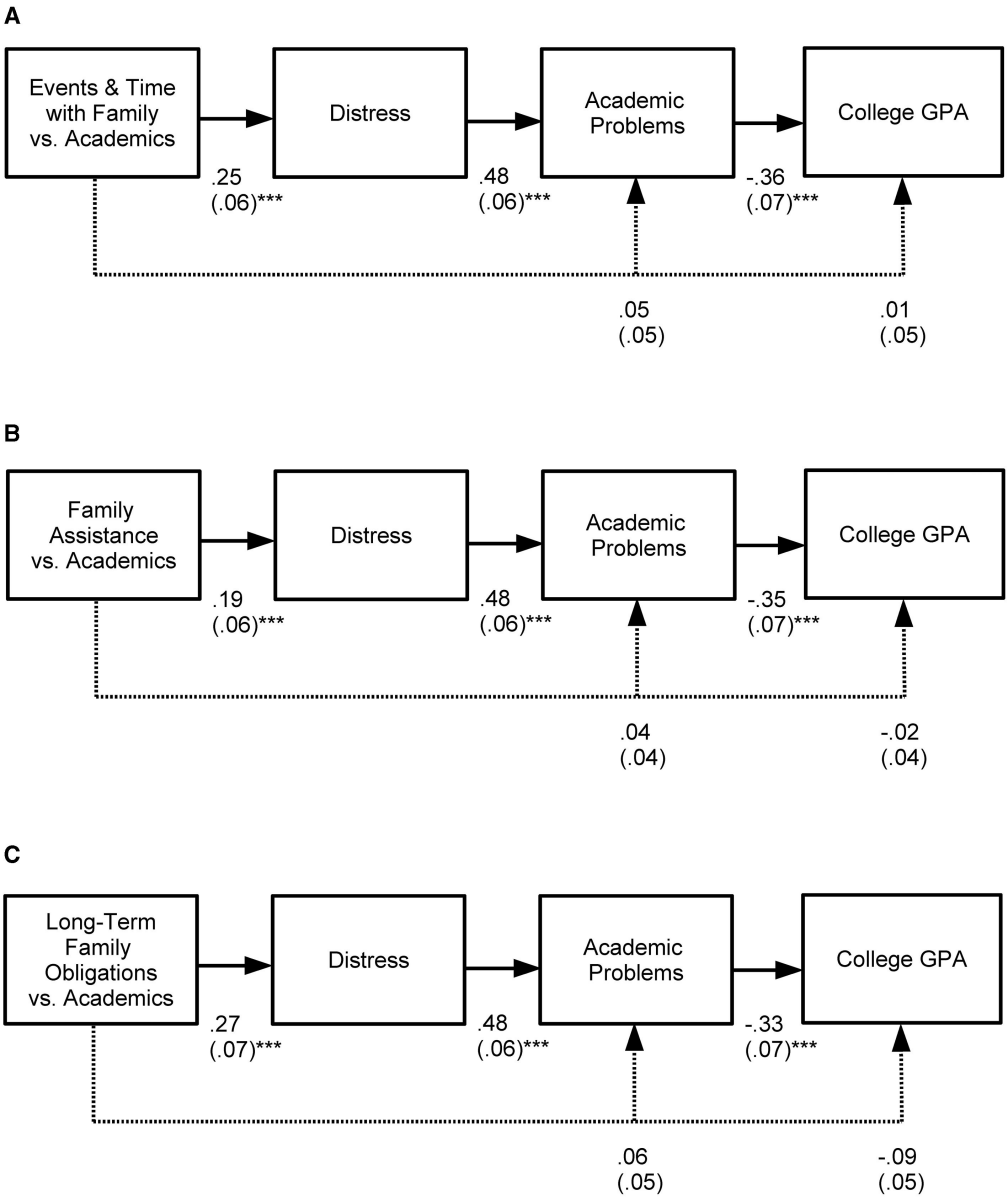
Consequences of the three forms of home-school cultural value mismatch: **(A)** events and time with family, **(B)** family assistance, and **(C)** long-term family obligations vs. academics. Included are unstandardized estimates (with standard errors); a solid line indicates significance and a dashed line indicates non-significance; ***p < 0.001.

These models examined consequences of home-school cultural value mismatch during the first year of college. Models fit the data well for each of the three aspects of home-school cultural value mismatch: events and time with the family [ χ^2^(1, *N* = 155) = 0.13, *p* = 0.720, CFI = 1.00, RMSEA = 0.000; Figure 4A]; family assistance [χ^2^(1, *N* = 155) = 0.25, *p* = 0.615, CFI = 1.00, RMSEA = 00; Figure 4B]; and long-term family obligations [χ^2^(1, *N* = 155) = 0.68, *p* = 0.409, CFI = 1.00, RMSEA = 0.00; Figure 4C]. Overall, 7–10% of the variance in mental and physical health distress, 34% of the variance in academic problems, and 16–18% of the variance in college GPA was explained.

**Hypothesis 6a**. Integrated into one large model, home-school cultural value mismatch will relate to mental and physical health distress (Figure 2). This relationship was confirmed for all three aspects of home-school value mismatch (Figures 4A–C).

**Hypothesis 6b**. Distress will, in turn, be associated with academic problems surrounding attention and learning (Figure 2). This relationship was also confirmed for all three aspects of home-school value mismatch (Figures 4A–C).

**Hypotheses 6c**. Academic problems will then be linked to low college GPA (Figure 2). This relationship was confirmed for all three aspects of home-school value mismatch (Figures 4A–C).

**Hypothesis 6d**. In addition, because we expect distress to serve as the main mediator that links home-school cultural value mismatch to academic problems, a direct link from mismatch to academic problems was not expected to be present within the model after accounting for distress (Figure 2). Instead, the hypothesis is that home-school cultural value mismatch will have a significant indirect effect on academic problems through distress (Figure 2).

As expected, there was no direct relationship between any of the three components of home-school cultural value mismatch and academic problems (Figures 4A–C) after accounting for distress. Instead, there were significant indirect effects for all components of home-school cultural value mismatch on academic problems (events and time with family: unstandardized indirect effect = 0.12; standardized indirect effect = 0.17; standard error = 0.03, *p* < 0.001; family assistance: unstandardized indirect effect = 0.09; standardized indirect effect = 0.15; standard error = 0.03, *p* < 0.001; long-term family obligations: unstandardized indirect effect = 0.13; standardized indirect effect = 0.17; standard error = 0.04, *p* < 0.001).

**Hypothesis 6e**. Lastly, because we expect distress and academic problems to serve as the main mediators that link home-school cultural value mismatch to GPA, a direct link from mismatch to GPA was not expected to be present within the model after accounting for distress and academic problems (Figure 2). Instead, the hypothesis is that home-school cultural value mismatch will have a significant indirect effect on GPA through the paths of the intervening variables (Figure 2).

As expected, there was no significant relationship between any of the three components of home-school cultural value mismatch and GPA (Figures 4A–C) after accounting for distress and academic problems. Instead, there were significant indirect effects for all components of home-school cultural value mismatch on college GPA (events and time with family: unstandardized indirect effect = −0.06; standardized indirect effect = −0.10; standard error = 0.02, *p* = 0.08; family assistance: unstandardized indirect effect = −0.05; standardized indirect effect = −0.09; standard error = 0.02, *p* = 0.015; long-term family obligations: unstandardized indirect effect = −0.06; standardized indirect effect = −0.09; standard error = 0.02, *p* = 0.007).

Finally, it is noteworthy to mention that our team tested several alternative models. For example, in terms of antecedents of home-school cultural value mismatch, we tested a model that situated Latinx and Asian American as a predictor, rather than Latinx alone. That model did not fit the data well. In terms of consequences, we tested a model that switched the variable order of distress and academic problems. That model did not fit the data well. Additionally, we tested a model that incorporated a direct link from distress to GPA; though this resulted in a saturated model, the path was not significant and other all paths continued to remain significant. Thus, the models reported in our manuscript worked best; they are also aligned with our theoretical frameworks and prior research.

## Discussion

Although research on disparities in educational attainment for Latinx youth has primarily focused on academic barriers, our quantitative findings suggest a different mechanism that explains Latinx students' difficulties during the first year of college: being a first-generation college student and experiencing *home-school cultural value mismatch*—mismatch between family and academic obligations. Students grappled with three sources of mismatch with their academic goals: (1) attending events and spending time with family, (2) assisting family in the here and now, and (3) choices between academics and family obligations with long-term implications. In a multi-ethnic sample in Los Angeles, three intercorrelated variables—Latinx ethnicity, first-generation college status, and low-income backgrounds—correlated significantly with higher levels of home-school cultural value mismatch. In a larger model, first-generation college status was the key variable that indirectly related to more experiences with all forms of home-school cultural value mismatch via a theoretically driven path involving cultural values—specifically collectivistic motives for attending college. In turn, all forms of mismatch were negatively related to health and academic development. Our documentation of the predictive antecedents and consequences of home-school cultural value mismatch provides quantitative support for prior qualitative findings (Vasquez-Salgado et al., 2015). It also provides a new line of evidence and a new perspective for Cultural Mismatch Theory (Stephens et al., 2012a) and the Theory of Social Change, Culture and Human Development (Greenfield, 2009).

Collectivistic motives for attending college are known to be in misalignment with the individualism of the university environment (Stephens et al., 2012a). In this study we show that such motives made students susceptible to all forms of mismatch between family and academic obligations. The nature of this mediation in the antecedents model, along with the correlation matrix (Table 1), implies that home-school cultural value mismatch is more prevalent among first-generation college students, who tend to report higher collectivistic motives for attending college. In addition, given the centrality of first-generation college status, we provide support for generalizing the experience of home-school cultural value mismatch to first-generation college students from multiple backgrounds, not just Latinx students (e.g., students from Asian, Black or Middle Eastern or Persian backgrounds who are first-generation college students; Greenfield, 2009; Stephens et al., 2012a; Vasquez-Salgado et al., 2015). At the same time, our findings demonstrate additional obligation for students from households with low parental income, particularly for mismatches involving choices between family and academic obligations that carry long-term imprints or weights.

The other sociodemographic variables in our antecedents model also played a significant role in students experiences with cultural value mismatch. As hypothesized, identifying as female was correlated with higher levels of all forms of home-school cultural value mismatch. This may be attributable to females having greater family obligations than males (Goodnow, 1988; Stein et al., 2014).

For geographical distance from home, there were some differences across the models. Living close to one's family home was predictive of more experiences with home-school cultural value mismatch, but only for mismatch related to attending events and spending time with family. This finding aligns with prior research suggesting that students living closer to home engage in more of these types of obligations (Tseng, 2004). Together, these findings demonstrate that the experience of home-school cultural value mismatch can be applied to students with other sociodemographic characteristics: females, and in some cases, students who live close to home. This is a unique contribution to Cultural Mismatch Theory and the Theory of Social Change, Culture and Human Development, as our findings go beyond variables pertaining to socioeconomic status (first-generation college status, income) and cultural values in predicting mismatch.

Results also confirmed our predicted model concerning the consequences of all forms of home-school cultural value mismatch. Home-school cultural value mismatch predicted mental and physical health distress. Distress, in turn, was related to a higher frequency of reported academic problems; more problems, in turn, were associated with lower GPAs during the first year of college. Additional findings confirmed that these linkages served as the main pathway linking cultural mismatch to academic problems, as well as cultural mismatch to lower GPA. This model, based on the broad notion that cultural mismatch may affect development (Greenfield, 2009; Stephens et al., 2012a) and grounded in the experiences of participants in a prior qualitative study (Vasquez-Salgado et al., 2015), provides the first quantitative support for an intrapersonal mechanism by which home-school cultural value mismatch relates to academic performance during the first year of college: mismatch → distress → academic problems → lower GPA. This model is unique in integrating mental and physical health with academic elements in a single model.

Note too that the psychometric analysis of this study provides some initial support for the construct validity of the three-factor home-school cultural value mismatch scale assessing mismatch between academics and (1) attending family events or spending time with family, (2) family assistance (e.g., taking care of other family members), as well as (3) mismatch between academics and family obligations that may have long-term consequences (e.g., making purchase for the family, living close to home) in a sample of undergraduates from diverse ethnic backgrounds. Though all forms of mismatch appeared to have contributed to health and academics, they slightly varied in models pertaining to the predictive antecedents. In addition, our more recent pilot work conducted with Latinx females suggests that though all forms of mismatch related to higher levels of perceived stress, only mismatch between academics and family obligations that may carry long-term imprints or weight related to a flat or blunted cortisol response upon waking^1^. A flat or blunted cortisol response upon waking is a biological indicator of stress (Saxbe, 2008) and associated with fatigue, burnout or exhaustion (Chida and Steptoe, 2009). Thus, our methodological and empirical findings regarding distinct sub-scales of home-school cultural value mismatch receive additional support. Nonetheless, given the moderate to strong correlations among the sub-scales, researchers may decide to examine them either as separate sub-scales or as one single measure of home-school cultural value mismatch; a similar practice has been carried out with other measures available in the field (e.g., family obligation; Fuligni et al., 1999).

### Contributions

The current study integrates Cultural Mismatch Theory (Stephens et al., 2012a) and the theory of Social Change, Culture, and Human Development (Greenfield, 2009) by including the role of first-generation college status, parental income and cultural values in one model. This study extends both theories by adding new sociodemographic variables—geographical distance from the family home and gender—as predictors of home-school cultural value mismatch. Empirically, this research goes beyond prior research on cultural mismatch in education by creating comprehensive models that spell out in detail both the sociodemographic antecedents of mismatch and the intrapersonal mechanism regarding its consequences.

Our work has identified the barrier of home-school cultural value mismatch in academic success among undergraduates from diverse cultural and socioeconomic backgrounds. Now that we have a foundational understanding of the mechanisms in antecedents and consequences of this mismatch, we believe future work can address these challenges and begin to leverage the cultural strengths of diverse communities of color (Yosso, 2005; Covarrubias et al., 2019). Our documentation of mechanisms and creation of a measure of home-school cultural value mismatch are an important step in this larger agenda.

### Limitations, Future Directions, and Implications for Institutional Change

Our study had four main limitations. First, the main dependent measure (home-school cultural value mismatch) was developed based on the experiences of one specific ethnic group (Latinx) and of one specific socioeconomic class (first-generation college students) and then applied to other ethnic groups (i.e., Asian and European Americans) and other social classes. Perhaps other home-school cultural value mismatches, not captured in our measure, would be relevant for youth from other ethnic groups or social classes. Nonetheless, the measure worked as predicted in our multi-ethnic sample, and psychometric analysis indicates the construct validity of the scale for capturing different sources of home-school cultural value mismatch.

Second, the ethnic groups in our sample varied in socioeconomic status. Latinx students constituted most of the low socioeconomic portion of our sample, and there were few first-generation college students among European Americans. Nonetheless, the socioeconomic disparities reflect the sociodemographic composition of most 4-year universities (Saenz et al., 2007) as well as the research site of this investigation (Guan et al., 2014). Thus, our sample has ecological validity.

Future research will replicate this study with a larger sample with an equal proportion of each of first- and continuing-generation students across various ethnic groups to allow for analysis of within-group variability in each ethnic group (García Coll et al., 1996). We have future plans to conduct this investigation at various universities across the country, as socioeconomic differences among Latinx, Asian and European American students likely vary across geographical contexts. For example, in Los Angeles, California, the Latinx population is predominately low in socioeconomic status; they are most often from Mexico and Central America. In contrast, the Latinx population in Miami, Florida is likely much more diverse in socioeconomic status; they are predominantly from Cuba. Similarly, in Los Angeles, California, the European American population is predominately high in socioeconomic status whereas the European American population in Ann Arbor, Michigan, is more diverse in socioeconomic status. Thus, our future designs will enable us to rigorously test the dimensional or intersectional nature of home-school cultural value mismatch, as well as how mismatch varies across geographical contexts.

Third, our study was conducted at a top-tier institution: UCLA. This is a very demanding educational context that makes it very difficult to harmonize family and academic obligations. This factor limits generalizability, as we may find less mismatch at less demanding postsecondary institutions. Indeed, prior research on cultural mismatch suggests that there is variation in individualistic and collectivistic norms that are expected of students across institutions (e.g., top-tier and second-tier universities), resulting in potential variation in cultural mismatch experiences (Stephens et al., 2012a). Future research will reexamine our findings across various types of institutional contexts.

Another limitation of our study was its cross-sectional nature. Though we used path analysis to document the mechanisms in antecedents and consequences of home-school cultural value mismatch, the cross-sectional nature of our study prevents us from claiming these mechanisms as a process that unfolds over time (Selig and Preacher, 2009). Thus, the links represented in our models represent a series of correlations that are consistent with but do not prove causal relations. We are currently working toward resolving this limitation by conducting a longitudinal investigation of cultural mismatch, health, and academics over time.

Lastly, and importantly, the current investigation has a larger, long-term goal of serving as a foundation for future work that will identify the cultural strengths and resilience against cultural mismatch and its negative impact on adjustment. There are several strengths that youth from communities of color, as well as those from low socioeconomic status households, bring with them in their transition to 4-year universities (Yosso, 2005; Covarrubias et al., 2019). Not all fit similar patterns of cultural mismatch and adjustment, and development does not unfold in one prototypical fashion (Rogoff, 2003; Greenfield, 2009; Vasquez-Salgado and Greenfield, 2021). However, when these sources of community cultural capital go unrecognized or are not valued within academic institutions, this omission can lead to marginalization of students of color and, ultimately, reduce the diversity of perspectives critical to economic and social advancement.

### Implications

Our findings have several implications for research, intervention, and larger, institutional understanding and change. In terms of research, our findings are the first to document the antecedents and consequences of cultural mismatch between the behavioral enactment of family obligations and academic obligations. Because we have shown that the key mechanism largely depends on sociodemographic factors and cultural values, rather than ethnicity, we can generalize the experience of home-school cultural value mismatch beyond Latinx students to students from other ethnic backgrounds.

Our findings also provide a psychometrically validated measure of home-school cultural value mismatch that may be used by other researchers in the scientific field. Expansion of knowledge in this area from a resilience and cultural strengths perspective will enable researchers to devise empirically-based interventions that may reduce cultural mismatch and the negative role it plays in overall adjustment. Such interventions may provide insight into ways that students can navigate these experiences.

In addition, given the current situation our society is facing with COVID-19 (also known as the novel coronavirus), the majority of students are engaging in virtual instruction from their parents' homes. Thus, our findings concerning the importance of geographical distance suggest a dire need for the development of online interventions for students who are first-generation college students, from low-income backgrounds, live at home, or identify as female. Prior research has shown that simply speaking about this mismatch in a focus group setting or listening to the voices of other students that have experienced general elements of mismatch may be beneficial (Stephens et al., 2014; Vasquez-Salgado et al., 2015). Thus, perhaps the topic of cultural mismatch between family obligations and academic obligations can be something that is discussed in current programs that are readily available to students.

Lastly, our findings can be used to help spawn larger, institutional change, such as making home-school cultural value mismatch explicit to students and other stakeholders of educational institutions. In terms of students, institutions can educate students to recognize the experience of home-school cultural value mismatch and its associated effects (e.g., at new student orientation, via online campaigns). This may help de-stigmatize the experience of this mismatch and create a culture where students feel that they can communicate their experiences of mismatch with their peers, professors and counselors. At the same time, it is crucial that all stakeholders in educational institutions, including individuals such as, deans, faculty, graduate teaching assistants, counselors and other student affairs staff (e.g., resident advisors, academic mentors, student orientation directors), be made aware of home-school cultural value mismatch. Communicating our findings and other cultural mismatch work to stakeholders may lead to the incorporation and sustainability of this topic as a focal point in current programs available to students. It may also lead to greater cross-cultural sensitivity. Together, these institutional practices may work to lessen the burden cultural mismatch places on students' health and academic development.

## Data Availability Statement

The dataset generated for this article is not available because our approved IRB (along with the consent forms signed by participants) states that the data must not be shared with anyone outside of our research team. Request for access to survey items, coding schemes or analytical procedures should be directed to yolanda.vasquez-salgado@csun.edu.

## Ethics Statement

The study involving human participants was reviewed and approved by the North General Institutional Review Board (NGIRB) from the College of Letters & Science and the Professional Schools at UCLA. The participants provided written informed consent to participate in this study.

## Author Contributions

YV-S and PG devised the study. YV-S gathered the data. YV-S and S-SG conducted the analyses. YV-S wrote the first draft of the manuscript. S-SG and PG contributed to the writing across the various versions. All authors approved the final version.

## Author Disclaimer

The content is solely the responsibility of the authors and does not necessarily represent the official views of the National Institutes of Health.

## Conflict of Interest

The authors declare that the research was conducted in the absence of any commercial or financial relationships that could be construed as a potential conflict of interest.

## Publisher's Note

All claims expressed in this article are solely those of the authors and do not necessarily represent those of their affiliated organizations, or those of the publisher, the editors and the reviewers. Any product that may be evaluated in this article, or claim that may be made by its manufacturer, is not guaranteed or endorsed by the publisher.

## Footnotes

^1^Vasquez-Salgado, Y., Guan, S. A., Toledo-Corral, C., and Serrano, J. (under review). Home-school cultural value mismatch and cortisol awakening response among Latinx females during the transition to college: A mixed methods analysis.

## Appendix A

**TABLE A1:** Principal components correlation matrix for home-school cultural value mismatch scale.

**Item Number**	**Item**	**Factor 1**	**Factor 2**	**Factor 3**
	Since you started UCLA, how often have you had to choose between your academic work and the following things?			
1	…attending family events.	**0.83**	−0.01	0.15
2	…attending cultural or religious family gatherings.	**0.76**	−0.23	0.22
4	…spending time with your parents or siblings on weekends.	**0.79**	0.20	−0.11
5	…spending time at home with family.	**0.78**	0.21	−0.10
6	…spending time with your grandparents, cousins, aunts, or uncles.	**0.72**	0.20	−0.13
7	…doing tasks your family needs done.	0.08	**0.77**	0.14
8	…helping out around your family's house.	0.22	**0.78**	−0.05
9	…helping taking care of family members (e.g., grandparents, parents, siblings).	0.05	**0.82**	0.07
10	…helping your brothers or sisters with their homework.	−0.06	**0.85**	0.00
3	…spending holidays with family.	0.19	0.11	**0.41**
	How often did it happen that you wanted to go further away from home, but your family wanted to following things?			
11	…for you to attend a college closer to home.	−0.28	0.30	**0.72**
12	…for you to continue living at home.	−0.15	0.25	**0.70**
	Since you started UCLA, how often have you had to choose between using your money for your college expenses (e.g., tuition, living costs) and the following things?			
13	…purchasing something for your family.	0.16	−0.16	**0.83**
14	…giving your family money for something they need.	0.19	−0.16	**0.83**

*Factor loadings in boldface are >0.30; Factor 1 = Events and time with family vs. academics; Factor 2 = Family assistance vs. academics; Factor 3 = Long-term family obligations vs. academics*.
